# Job-related stress associated with work-related upper extremity musculoskeletal disorders (UEMDs) in municipal waste collectors: the moderation and mediation effect of job support

**DOI:** 10.1186/s12891-022-05721-y

**Published:** 2022-08-10

**Authors:** Ching-Lan Yang, Wei-Ping Huang, Wen-Yu Lin, Po-Chang Tseng, Hsien-Wen Kuo

**Affiliations:** 1 Occupational Medicine Department, Far Eastern Memorial Hospital, Taipei, Taiwan; 2 Institute of Environmental and Occupational Health Sciences, National Yang Ming Chiao Tung University, 155, Sec.2, Linong Street, 112 Taipei, Taiwan; 3 Health Promotion Administration, Ministry of Health and Welfare, Taipei, Taiwan; 4 Institute of Public Health, National Defense University, Taipei, Taiwan

**Keywords:** Job-related stress, Job support, Work-related upper extremity disorders, Municipal waste collectors

## Abstract

**Background:**

Since the policy of “keeping trash off of the ground” in Taiwan, long-term exposure to repetitive motion in waste collection process results in high risk of upper extremity musculoskeletal disorders (UEMDs). Thus, we assessed the moderation and mediation effects of job-related stress and job support on work-related UEMDs among municipal waste collectors.

**Methods:**

A cross-sectional study was conducted in two cities located at northern Taiwan during 2018–2019. 626 municipal waste collectors voluntarily participated and anonymously filled out a structured questionnaire. The moderation and mediation effects of effort-reward imbalance (ERI) and social support on UEMDs were analyzed by Haye’s Process Macro Model.

**Results:**

Prevalence of UEMDs in municipal waste collectors were 43.4% for neck, 56.0% for shoulder, 24.1% for upper back, and 33.1% for hand/wrist. There was high prevalence of shoulder (72.2%), neck (48%), and upper back (30%) in female workers compared to male, most significantly in shoulders. In univariate and multivariate analysis, high ERI and low job support were significantly associated with ORs of 3.11 (95% CI:1.58–6.13) for elbow, 2.79 (95% CI:1.39–5.56) for shoulder, 3.39 (95% CI:1.64–7.00) for upper back and 3.83 (95% CI:1.98–7.41) for hand/wrist. Prevalent UEMDs were positively associated with high ERI in municipal waste collectors but negatively with job support. The moderation effects of ERI and job support on UEMDs, of which the measured synergy index exceeded one, were 18.24 for shoulder, 3.32 for elbow, and 2.45 for hand/wrist, but mediation effects were not significant.

**Conclusions:**

This study found municipal waste collectors with work-related upper extremity disorders were significantly associated with work-related psychological risk factors. Therefore, waste collection cannot only to be improved by semi-automatic and automatic processes but immediate intervention programs for the reduction of psychological risk factors is needed promptly.

## Background

Annual growth of municipal waste in Taiwan’s cities have averaged 5% -7% due to rapid urbanization and economic development. In 2017, 130,735 tons of refuse for disposal and 4,113,808 tons of recycled recyclable waste were generated [1]. Based on the policy of “keeping trash off of the ground” in Taiwan, each citizen is required to make stops to throw their trash directly into a trash truck at designated times and locations rather than leaving it on the ground randomly. Although some citizens still complain about having to come out at specific times to dispose of their garbage, most citizens have become remarkably cleaner as a result of the policy [2]. As a result, the collection of household waste among municipal waste collectors (MWCs) requires repeated heavy physical activity such as lifting, carrying, pulling, and pushing, which significantly increased incidents of musculoskeletal disorders (MSDs) [3]. Municipal waste collectors (MWCs) need to help citizens to quickly throw various solid waste into the inside of trash truck, which has short stays at each location. In addition, they are also charged with the responsibilities of handling recyclable items from municipal waste and periodically cleaning street roads in their city. Subsequently, the MWCs in Taiwan characteristically has a heavier workload than waste pickers who typically salvage recyclable items and collect rubbish (paper, plastic, tin and so on). Since waste collecting varies from informal manual to semi-automated systems, workers are frequently exposed to high work demands and/or acute bodily responses, such as musculoskeletal disorders [4, 5]. About 40% of the municipality solid waste collectors in Iran were exposed to high physical and psychological workloads. A study [6] in Iran indicated that 92.5% of waste collectors self-reported MSD symptoms in at least one body region during the last 12 months. Similarly, the health effects among MWCs in Taiwan in particular, had a high prevalence of lower back pain, elbow and wrist pain, and injuries caused by sharp objects [7]. Obviously, MWCs who frequently suffer from upper extremity musculoskeletal disorders (UEMDs) have become an increasingly significant proportion of compensation claims, disorders which include muscles, tendons, or nerves, of the shoulder, arm, elbow, wrist, or hand [8]. A meta-analysis from twenty-four articles [9] has indicated that hospital nurses and nursing aides with high psychosocial demands and low job control are significantly associated with MSDs. While risk factors from demographic characteristics was not controlled by medical intervention, exposure to work-related factors can modified by adjustment of work process, e.g. waste collection. So far, previous studies focused on the effect of physical workloads on MSDs, few studies have examined the moderation and mediation effects of work-related psychosocial factors, included job-related stress and social support on UEMDs. Consequently, the aim of our study was to evaluate the moderation and mediation effects of job-related stress and job support on UEMDs in MWCs.

## Methods

### Study population

622 waste collectors (including drivers, waste collectors, and others) voluntarily enrolled and participated from two cities (Keelung city and Yilan county) in northern Taiwan between March 2018 and July 2019. All participants provided written consent prior to the study, and the study was approved by the institutional review board of the Yang Ming University. The overall response rate was 83%. The reasons for non-response included vacations, requested time off, or having limited time to fill out the questionnaire, and they do not affect the objective of the study.

### Measurement

Each employee signed an informed consent at the time of the study and anonymously filled out the web-based questionnaire that included demographic data, job characteristics (job title, job schedule, and work duration), psychological risk factors (ERI, social support), and musculoskeletal disorders (MSDs). ERI was measured by a short-modified questionnaire [10], which contained three items for measuring effort and 7 items for measuring reward. The ERI ratio for each study participant was computed using the sum scores for efforts as the numerator (Effort) and the sum scores for rewards as the denominator (Reward) multiplied by a correction factor of 0.43 [(Effort/3) / (Reward/7)]. An ERI ratio≧1 indicated an exposure to a high ERI at work, which constituted as a perceived job-related stress. Cronbach's alpha coefficients for effort and reward were 0.74 and 0.83, respectively, which is consistent with Siegrist’s study (1996) of 0.61 -0.91. Job support (JS) was measured by eight items including work support and guidance by colleagues and superiors [11]. The Likert scale was used to measure JS: strongly agree (4 points), agree (3 points), disagree (2 points), and strongly disagree (1 point). Higher scores represented employees with higher JS levels in their workplace. Three specialists including an occupational physician, an epidemiologist, and an occupational hygienist have reviewed the content validity of the questionnaire. The reliability test for JS measured by Cronbach's α was 0.93. Lastly, burnout levels were measured by a 5-item Chinese-version questionnaire [12]. The Likert scale was used to measure work-fatigue: always (3 points), usual (2 points), occasional (1 point), and never (0 points).

### Outcome measurement

Upper extremity musculoskeletal disorders (UEMDs) were defined as symptoms of numbness or pain in the upper extremity location including neck, shoulder, upper back, elbows, and hand or wrist over the previous year using the Chinese version of the Nordic musculoskeletal questionnaire (NMQ) [13]. Each MWC with UEMDs self-reported the severity of symptoms and treatment in previous year. The average of content validity index in web-questionnaire was 0.91, which is verified by five experts.

### Statistical analysis

The SPSS 24 package (SPSS Inc, Chicago, IL, USA) was used to perform descriptive analysis for demographic data, which are expressed as percentage. The Chi-square test was used the distribution of UEMDs prevalent and gender. Scores for social support were classified into high (≧75% of total scores) and low (< 74% of total scores) groups. The odds ratios (ORs) with lower (LCL) and upper confidence limit (UCL) in the high and low groups of social support or ERI examined the association with UEMDs using multivariate analysis after adjusted for covariates. Synergy index was used to assess the interaction effect of ERI and social support using dichromatic scale on UEMDs. The mediation model was analyzed using Haye’s model 4 based on the PROCESS macro for SPSS [14]. The bias-corrected 95% confidence limit was calculated with 5000 bootstrapping re-samples. The association between work-related stress and UEMDs mediated by social support was also tested. All p values were two-sided, and all results were considered statistically significant at p < 0.05.

## Results

### Demographics and prevalence of UEMDs in municipal waste collectors

Table 1 shows demographics in municipal waste collectors (MWC). Results indicate 85% for male, 60% age between 36–55 years, 69% married, and 28% with only a junior high education, and 95% with a monthly income less than 50,000NTD. In regards to job characteristics, 75% of participants were waste collectors, 73% had worked the job for less than 20 years, and 95% were on a fixed work schedule. Comparison of prevalence rates of UEMDs in male and female waste collectors is shown in Table 2. The prevalence rate of shoulder disorders in female workers (72%) was significantly higher than in male workers (53%). There was slightly higher prevalence of upper back problems (30%) in female compared to male workers, but it was insignificant.

**Table 1:** Demographics and job characteristics in municipal waste collectors (*N* = 622)

	*n*(%)
Gender	
Male	525(85.2)
Female	91(14.8)
Age (years)	
≦35	94(15.3)
36–55	367(59.9)
≧56	152(24.8)
Education	
≦Junior high	175(28.3)
Senior high	254(41.1)
≧College	189(30.6)
Job title	
Collectors	467(75.3)
Driver	81(13.1)
Other	72(11.6)
Work duration (months)	
≦24	54(14.9)
25–239	208(57.5)
≧240	100(27.6)
Work schedule	
Fixed	581(94.3)
Non-fixed	35(5.7)
Marital status	
Single	124(20.2)
Married	422(68.8)
Other	67(10.9)
Income (NTD/month)	
≦50,000	573(95.0)
> 50,000	30(5.0)

**Table 2:** Prevalence of upper extremity musculoskeletal disorder (UEMDs in male and female waste collectors)

	Male (*N* = 499)	Female (*N* = 90)	Total (*N* = 589)	*p*-value
Elbow	138(27.7)	25(27.8)	163 (27.6)	0.981
Shoulder	265(53.1)	65(72.2)	330(56.0)	< 0.001
Upper back	115(23.0)	27(30.0)	142(24.1)	0.156
Hand/wrist	166(33.3)	29(32.2)	195(33.1)	0.846

### Determinants of UEMDs

The association of social support and ERI with the prevalence of UEMDs was examined using logistic regression adjusted for education, job title, marital status, work duration, and income in Table 3. In multivariate analysis, UEMDs were only significantly lower in upper back (OR = 0.45, 95% of CI: 0.26–0.79) and hand/wrist (OR = 0.48; 95% of CI: 0.29–0.79)) in the high social support group. In sum, the results indicated that a reduction of 21% to 55% of UEMDs were found in the high social support group. Summarily, there were consistently high ORs of UEMDs in the ERI ≧ 1 group. Using multivariate analysis after adjusting for covariates, MCWs with high job-related stress had high ORs of UEMDs, including 2.38 (95% of CI: 1.42–4.01) for the elbow, 1.70 (95% of CI: 1.06–2.74) for the shoulder, 2.27 (95% of CI: 1.30–3.95) for the upper back, and 2.40 (95% of CI: 1.45–3.95) for the hand/wrist, respectively. Results indicated MCWs with high job-related stress had significantly high ORs in UEMDs, especially for upper back and hand/wrist locations.

**Table 3:** Social support and ERI associated with the prevalence of UEMDs using logistic regression after adjusting for education, job title, marital status, work duration, and income

	Low JS (*N* = 166) *n*(%)	High JS (*N* = 428) *n*%	AOR	95%LCL	95%UCL
Elbow	54(32.5)	111(25.9)	0.63	0.37	1.08
Shoulder	106(63.9)	226(52.8)	0.63	0.38	1.03
Upper back	58(34.9)	85(19.9)	0.45**	0.26	0.79
Hand/wrist	59(41.6)	127(29.7)	0.48**	0.29	0.79
	ERI < 1 (N = 400) *n*(%)	ERI≧1 (N = 194) *n*%	AOR	95%L	95%U
Elbow	95(23.8)	70(36.1)	2.38**	1.42	4.01
Shoulder	209(52.2)	123(63.4)	1.70*	1.06	2.74
Upper back	78(19.5)	65(33.5)	2.27**	1.3	3.95
Hand/wrist	116(29.0)	80(41.2)	2.4**	1.45	3.95

^*^p-value < 0.05;

^**^p-value < 0.01

### Moderation effects of social support and ERI on UEMDs

Table 4 illustrates the moderation effects of social support and ERI on UEMDs using logistic regression adjusted for education, job title, marital status, work duration, and income. The group with both high ERI and low social support had significantly high ORs in UEMDs, revealing 3.11 (95% of CI: 1.58–6.13) for elbow, 2.79 (95% of CI: 1.39–5.56) for shoulder, 3.39 (95% of CI: 1.64–7.00) for upper back, and 3.83 95% of CI: 1.98–7.41) for hand/wrist, respectively. Compared to the group with low ERI and high social support, there were only significantly high scores in the three other groups on upper back disorders, including 2.37 (95% of CI: 1.05–5.35) for in the group with low ERI and low social support, 2.38 (95% of CI: 1.13–5.01) for the group with high ERI and high social support, respectively. Results showed that moderation effects using synergy index exceeding one were 3.32 for elbow disorder, 18.24 for shoulder disorder, and 2.45 for hand/wrist disorder, but it was not found in the upper back disorder.

**Table 4:** Moderation effects of ERI and social support on UEMDs using logistic regression adjusted for education, job title, marital status, work duration, and income

		Elbow	Shoulder	Upper back	Hand/wrist
ERI	Social support	OR(95%CI)	OR(95%CI)	OR(95%CI)	OR(95%CI)
Low	High	1	1	1	1
Low	Low	0.88 (0.38–2.04)	0.95 (0.49–1.87)	2.37* (1.05–5.35)	1.39 (0.65–2.98)
High	High	1.76 (0.89–3.45)	1.14 (0.63–2.08)	2.38* (1.13–5.01)	1.77 (0.91–3.43)
High	Low	3.11** (1.58–6.13)	2.79** (1.39–5.56)	3.39** (1.64–7.00)	3.83** (1.98–7.41)
Synergy index		3.32	18.24	0.87	2.45

^*^p < 0.05;

^**^p < 0.01

**Fig. 1:**
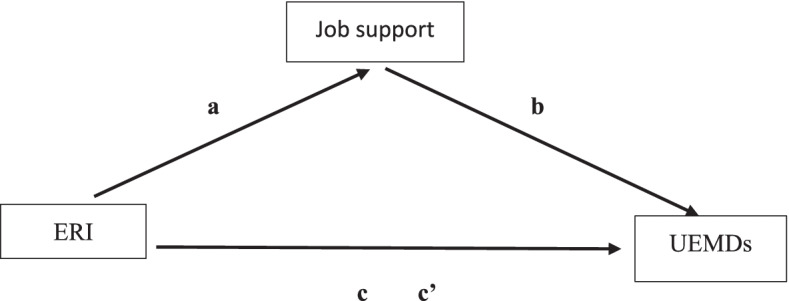
Schematic of the mediation effect of job support on the association between ERI and UEMDs using Haye’s Model 4. a: ERI lead to the effect of job sipport; **b**: job support affects the effect of UEMDs; **c**: ERI directly affects to UEMDs; **c**’: Indirect effect of job support on the association between ERI and UEMDs

Figure 1 indicates the mediation effect of job support on the association between ERI and UEMDs using Haye’s Model 4. Job support was assumed to be a predictor of ERI and UEMDs. Table 5 indicates the mediation effects of job support on the relationship between ERI and UEMDs using Haye’s model 4. A significant association was found between the direct effects of ERI on four locations of UEMDs, the highest effect was on shoulder disorders and the lowest effect on elbow disorders. The percentages of indirect effects contributing from job support on the association between ERI and UEMDs found was a considerably small range from 5.8% to 15.5%, but none are significant.

**Table 5:** Mediation effects of job support (JS) on the relationship between ERI and upper extremity musculoskeletal disorders (UEMDs) using Haye’s model 4

UEMDs	Direct effect of ERI on UEMDs	Indirect effect of JS on ERI and UEMDs	Percentage of indirect effect
	ß (SE)	ß (SE)	
Elbow	0.869**(0.343)	0.133(0.194)	13.3%
Shoulder	1.776**(0.400)	0.325(0.181)	15.5%
Upper back	1.195**(0.374)	0.203(0.208)	14.5%
Hand/wrist	1.249**(0.354)	0.077(0.182)	5.8%

^**^p < 0.01

## Discussion

### Ergonomic factors and MSDs

Since Taiwan’s Environmental Protection Agency (EPA) prohibited randomly throwing away municipal solid waste, MWCs frequently assist citizens in quickly throwing garbage into vehicles, manually operating the handling and transportation of waste in short amounts of time. Unsurprisingly, the waste collection process is characterized by the workplace 3Ds dangerous, drudging, and demanding, enhancing the potential individual risk of occupational morbidities and injuries [15]. Upper extremity musculoskeletal disorders (UEMDs) are an ergonomic-related occupational disease and place huge burdens on global workforces, which account for over 45% of all occupational diseases in Europe [16]. Based on observational interviews of the waste collection process from three experts, ergonomic hazards were assessed by key indicator method (KIM) in three domains including manual handing operations (KIM-HMO), lifting/holding/carrying (KIN-LHC) and pulling/pushing (KIM-PP) [17]. In our study, MWCs were at a moderate ergonomic risk (10–25 scores) as evaluated by the KIM tool. Repetitive and redundant operation in waste collection may cause adverse health effects for vulnerable groups. New ergonomic standards are needed to act as reference for interactions between job and machinery designers and ergonomists in the design of work processes and workplaces to reduce risk of UEMDs for repetitive activities [16].

### Prevalence and influencing factors of MSDs

The overall prevalence rates of MSDs among waste cleaners were approximately 90% and occurred in at least one body site, most prevalent in hand/wrist (42%), shoulder (41%), and lower back (38%) [18]. Employees manually operating waste collection frequently contributed to high incidence of MSDs, especially for UEMDs [15, 18]. A study [19] indicated that 56% of grinders and packers experienced at least one UEMD at the elbow, forearm, and/or wrist, mainly with signs of epicondylitis and nerve entrapment at the medial elbow. Although the risk of clinically relevant DASH (disabilities of the arm, shoulder, and hand) is > 29, grinders were with 2.5-times and packers 8.6 times more likely to develop UEMDs, these differences were not statistically significant. Another study [20], prevalence of pain in either neck, shoulder, or both sites in the previous year was 66.6% in 422 tailors, including 72.1% in shoulder and 68.3% in neck, respectively. Physical factors of the work environment significantly associated with factors of neck and/or shoulder pains included work experience, rest break, awkward working posture, prolonged sitting, and inadequate light. Physical factors of tailors in the workplace were better than those in MWCs who usually perform monotonous and repetitive jobs involving prolonged lifting and frequent movements of the upper extremities. Consistent with a study for steel workers from Aghilinejad et al. [21], working posture (bending and/or twisting) has been recognized as a significant risk factor for neck and shoulder disorders. In addition, ergonomic risk factors such as awkward posture, repetitive motions, and forceful exertion frequently occur in the waste collection process. Similar to other highly repetitive work tasks in another study [22], a high prevalence rate (35%) of neck or shoulder disorder was found among women in the fish processing industry, attributed to muscular tension, stress or worry, work strain, and frequent upper extremity use during the work of fish processing. In Ohlsson’s study [23], there were statistically significant associations between exposure to repetitive work and both neck/shoulder disorders (prevalence odds ratio, POR = 4.6) and elbows/hands (POR = 3.5). Also, low muscle strength, lack of emotional well-being at work, and a variety of psychosomatic symptoms were associated with diagnoses in the neck/shoulders. Therefore, implementation of multi-faceted ergonomic interventions [24], which consisted of imparting knowledge and training about ergonomics, workstation modification, training and surveying ergonomics at the workstation, and a regular exercise program into the workplaces is crucial to eliminate environmental hazards and eventually reduce risk of MSDs, especially for the upper extremities.

### ERI and social support associated with UEMDs

A study [21] indicted workers with low muscle strength, lack of emotional well-being at work, and a variety of psychosomatic symptoms were associated with diagnoses in the neck/shoulders. Since UEMDs are influenced by individual, environmental, psychosocial, and organizational factors, these disorders occurred under the impact or interaction of various long-term factors exceeding worker’s capacity. Notably, our findings highlighted that repetitive work with garbage collection were associated with a higher risk of UEMDs in MWCs, but psychological factors cannot be ruled out. The events of UEMDs also can be contributing to the moderation and mediation effects of ERI and social support. In the study, Table 3 indicted reductions of 21% to 55% of UEMDs in the high social support group but consistently high ORs (2.38 for elbow, 1.70 for shoulder, 2.27 for upper back, and 2.40 for hand / wrist, respectively) of UEMDs in the ERI ≧ 1 group. In addition, we examined the interaction effect of ERI and social support on UEMDs, of which the synergy index was 3.32 for elbow, 18.24 for shoulder, and 2.45 for hand/wrist locations. In mediation analysis, the percentages of indirect effects from job support on ERI and UEMDs were found in the range of 5.8% to 15.5% but none were significant. Importantly, job-related stress may independently and jointly with a lack of job social support contribute to prevalent UEMDs. Notably, MWCs with high ERI and low social support from colleagues and managers had high likelihood of UEMDs in the waste collection process. Excessive physical demands can overload the upper extremities, resulting in tissue breakdown and leading to physical symptoms such as elbow, hand/wrist, shoulder, or upper back pain. Our results are consistent with previous studies [25, 26] that have shown psychosocial factors are associated with the risk of both stress and MSDs. The psychosocial factors associated with the occurrence of MSDs are explained by psychological stress and or work strain, subsequently leading to adverse changes in immune system response, or even changes in adrenaline or noradrenaline [27]. An initiation of interventions aimed at tackling stress and MSDs might be more effective in improving the health of individuals, and as a result, that of organizations through reductions of job stress, awareness of MSDs, and increased productivity and reduced absence rates from MSDs through the modification of workstations.

### Strengths and limitations

Although the specific route by which job-related stress might cause risk of MSDs is a matter of debate, we propose both one-directional and mutual causality, that a lack of social support and job stress leads MSDs, as well as MSDs increasing job stress compounds into even more severe physiological symptoms. This is the first study in Taiwan explore the moderation and mediation effects of psychosocial stress models on UEMDs among MWCs. Our results also demonstrated that job-related stress measured by ERI independently or jointly contributes to more UEMDs. Consequently, based on the views of occupational health practices, we notified managers in waste collection processes to the significance of reducing effort, increasing rewards, and social support from encouragement, sponsorship, or resources provided by the organization, teamwork and colleagues. However, the study had several limitations. Since our study is a cross-sectional design, we cannot establish causality between job-related stress and social support with the occurrence of UEMDs. MWCs who may have retired early or been laid off with late-stage disorders that seek medical attention were not included, the result may lead to healthy worker effect and an underestimation of the association of the disease. In addition, our results need to examine the consistency between study results from both self-administered questionnaires and face-to-face physical assessments, especially regarding severity and clinical diagnosis of UEMDs among MWCs. A cross-sectional study [22] used self-reported questionnaires and pathognomonic clinical signs (trigger finger, Finkelstein’s test, Maudsley’s test, Hoffman–Tinel sign, and Phalen’s test) to evaluate upper extremity pain with different results; however, they also concluded that the questionnaire approach gives a good picture of the upper extremity locations with the possibility that the questionnaire can also underestimate severity. In addition, job-related stress in workplace has been accurately assessed by biological markers including saliva and urinary cortisol secretion, which are triggered by the hypothalamic–pituitary–adrenal (HPA) axis under stress and results in cortisol secretions intended to regain homeostasis [28, 29]. Further research is needed to verify job-related stress levels by ERI compared to assessment by biological markers.

## Conclusions

The moderation effects of effort-reward imbalance and job support on upper extremity musculoskeletal disorders were found, but the mediation effects were non-significant among municipal waste collectors. Our results recommended that the process of waste collection not only provides alternative equipment and modification of work schedule, but we proactively cooperate and internally communicate the role of job-related stress and upper extremity musculoskeletal disorder for reduction of psychological risk factors for municipal waste collectors.

## Data Availability

The data was collected and created anonymously among municipal waste collects on the two cities. The two city governments have agreed to participate in the survey and will be taken as a reference to modify the current work process of waste collection. Meanwhile, our findings were publicly accessible and freely available.

## Abbreviations

UEMDs: Upper extremity musculoskeletal disorder
MCWs: Municipal waste collectors
ERI: Effort-reward imbalance
MSDs: Musculoskeletal disorders
ORs: Odds ratios
LCL: Lower confidence limit
UCL: Upper confidence limit
